# Anjiang formula inhibits PVN microglial activation and lowers blood pressure by targeting RhoA/ROCK2 pathway: a retrospective clinical and experimental study

**DOI:** 10.3389/fphar.2026.1795297

**Published:** 2026-02-19

**Authors:** Xuanjing Chen, Jun Chen, Zhenglin Chen, Dawei Lian, Jing Wang, Meizu Wu, Jin Luo, Aling Shen, Lianfa Chen

**Affiliations:** 1 Department of Cardiology, Xiamen Traditional Chinese Medicine Hospital, Xiamen, China; 2 First Clinical Medical College, Fujian University of Traditional Chinese Medicine, Fuzhou, China; 3 Academy of Integrative Medicine, Fujian University of Traditional Chinese Medicine, Fuzhou, Fujian, China; 4 College of Integrative Medicine, Fujian University of Traditional Chinese Medicine, Fuzhou, Fujian, China

**Keywords:** microglial activation, neuroinflammation, oxidative stress, paraventricular nucleus, ROCK2 signaling, translational medicine, vascular remodeling

## Abstract

**Background:**

Neuroinflammation in the hypothalamic paraventricular nucleus (PVN) drives sympathetic overactivity in hypertension. The Anjiang Formula (AJ) shows clinical antihypertensive potential; however, the precise molecular targets mediating its central neuroprotective effects remain undefined.

**Methods:**

In this translational study, we investigated the clinical efficacy of AJ and tested the hypothesis that it directly inhibits the central RhoA/ROCK2 signaling axis. We integrated a retrospective cohort analysis with mechanistic validation. Clinically, 85 elderly patients with Grade 1 essential hypertension were treated with AJ (*n* = 43) or lifestyle control (*n* = 42) for 8 weeks. Target engagement was verified using surface plasmon resonance (SPR), microscale thermophoresis (MST), and cellular thermal shift assays (CETSA). Mechanisms were validated in Spontaneously Hypertensive Rats (SHRs) and Angiotensin II-stimulated microglia.

**Results:**

Clinically, AJ reduced systolic blood pressure (SBP) by a mean difference of 10.2 mmHg compared to controls (*p <* 0.001), with a 93% responder rate. This was accompanied by improved flow-mediated dilation (+1.3%) and reduced serum IL-6. Biophysical assays identified Shinflavanone as a direct ROCK2 ligand (*K*_D_ = 20.0 *n*M; CETSA Δ*T*_*m*_ = +5.2 °C). In SHRs, AJ lowered blood pressure and suppressed PVN microglial activation. *In vitro*, AJ inhibited the RhoA/ROCK2 cascade, downregulated JUN, and upregulated CREB1/NQO1, thereby reducing oxidative stress. These effects were abolished by the ROCK2 agonist lysophosphatidic acid.

**Conclusion:**

AJ provides antihypertensive efficacy in elderly patients. These benefits are mechanistically driven by Shinflavanone-mediated inhibition of ROCK2, which attenuates central neuroinflammation and restores redox homeostasis in the PVN.

## 1 Introduction

Hypertension remains the leading modifiable risk factor for cardiovascular mortality and morbidity worldwide, affecting over 1.3 billion adults (NCD Risk Factor Collaboration NCD-RisC, 2021). Despite the widespread availability of pharmacological agents targeting the renin-angiotensin-aldosterone system (RAAS) and calcium channels, a substantial proportion of patients fail to achieve optimal blood pressure control, leading to a phenomenon known as “residual cardiovascular risk (Whelton et al., 2017).” This persistent risk drives target organ damage, particularly hypertensive encephalopathy, cardiac hypertrophy, and renal fibrosis. Recent paradigm shifts in hypertension research have increasingly focused on the “Brain-Heart Axis,” identifying central nervous system (CNS) dysfunction—specifically sympathetic overactivity—as a critical driver of resistant hypertension (Xu et al., 2023; Nguyen et al., 2024). Consequently, identifying novel therapeutic targets that can breach the blood-brain barrier to modulate central sympathetic outflow represents a frontier in precision cardiovascular medicine.

The hypothalamic paraventricular nucleus (PVN) serves as a critical integration center for autonomic regulation. Emerging evidence from single-cell transcriptomics and high-resolution imaging implicates neuroinflammation within the PVN as a hallmark of hypertensive pathology (Goncharuk, 2021). Under hypertensive stimuli, such as elevated central Angiotensin II (AngII), microglia—the resident immune sentinels of the CNS—undergo a phenotypic transition from a “surveillance” state to a pro-inflammatory “activated” state (Wang et al., 2022). This maladaptive activation triggers an oxidative storm characterized by the assembly of NADPH oxidase (NOX) complexes and the release of reactive oxygen species (ROS) and cytokines (e.g., IL-6, TNF-α) (Baumer et al., 2025). This “oxidative-inflammatory” cycle in the PVN creates a feed-forward loop that tonically excites pre-sympathetic neurons, perpetuating systemic vasoconstriction and end-organ damage (Paul et al., 2022). Thus, dampening PVN microglial activation offers a strategic upstream approach to interrupt the maintenance of hypertension. Despite this mechanistic clarity, clinically viable agents that effectively suppress central neuroinflammation without compromising systemic immunity remain elusive. The RhoA/ROCK (Rho-associated coiled-coil containing protein kinase) signaling pathway has emerged as a pivotal “molecular switch” governing microglial cytoskeletal rearrangement and oxidative stress (Glotfelty et al., 2023). Specifically, the ROCK2 isoform regulates the morphological transition of microglia (from ramified to amoeboid) and facilitates the phosphorylation of subunits required for ROS generation (Zhang et al., 2014). While synthetic ROCK inhibitors exist, their clinical utility is often limited by systemic off-target effects. Therefore, the discovery of small molecules capable of selective central ROCK2 inhibition could revolutionize neuro-cardiovascular therapeutics.

Natural products have historically served as a rich reservoir for cardiovascular drug discovery. The Anjiang Formula (AJ), a clinically utilized antihypertensive Botanical drug prescription, was developed on the basis of the clinical experience of Academician Chen Keji and is guided by the TCM principle of “the heart governing mental activities” (Zhang et al., 2025). The formula comprises Semen Ziziphi Spinosae (Suanzaoren), Tall Gastrodiae (Tianma), Common Achyranthes (Niuxi), Poria (Fushen), White Peony Root (Baishao), Chinese Thorawax Root (Chaihu), Submature Bitter Orange (Zhike), and Licorice Root (Gancao). Preliminary clinical research suggests that AJ lowers blood pressure, improves serum neurotransmitter profiles and modulates the RAAS (Makuch-Martins et al., 2024). However, the specific molecular mechanisms underlying the antihypertensive effects of AJ remain to be systematically elucidated.

In this study, we employed a translational “bedside-to-bench” strategy to systematically deconvolute the antihypertensive efficacy and precise molecular mechanism of the AJ. Integrating a retrospective clinical analysis with advanced structural biophysics, we aimed to identify the direct therapeutic target of AJ and validate its physical engagement. Furthermore, utilizing hypertensive animal models and microglial systems, we sought to test the hypothesis that AJ functions as a central modulator of the RhoA/ROCK2 signaling axis, thereby arresting the oxidative-inflammatory cycle within the hypothalamus and normalizing sympathetic outflow.

## 2 Materials and methods

### 2.1 Clinical study design

This study employed a retrospective cohort design utilizing archived biological samples and clinical data from a randomized controlled trial (RCT) evaluating the efficacy of the AJ in elderly patients (≥60 years) with Grade 1 essential hypertension (SBP ≥140 mmHg and DBP ≥90 mmHg) (Writing Group of Chinese Guidelines for Hypertension Prevention and Control, 2024). The original trial was conducted at *Xiamen Traditional Chinese Medicine Hospital*. For the current analysis, 85 participants were selected based on the strict availability of paired blood samples at baseline and the 8-week follow-up. The study protocol was approved by the Institutional Review Board (IRB) of *Xiamen Traditional Chinese Medicine Hospital* (Approval No. XMTCM-2024.003) and strictly adhered to the principles of the Declaration of Helsinki. Informed consent for the secondary use of stored biological samples and anonymized data was obtained from all participants upon initial enrollment.

### 2.2 Clinical data acquisition and biomarker profiling

Primary clinical outcomes, including SBP and DBP, were measured using an automated oscillometric device (Omron HEM-907XL). The mean of the last two of three consecutive measurements was recorded. Vascular function was assessed via Flow-Mediated Dilation (FMD), Pulse Wave Velocity (PWV), and Carotid Intima-Media Thickness (cIMT) following standardized ultrasound protocols. Left Ventricular Mass Index (LVMI) was determined via echocardiography. Biomarker quantification was performed on serum samples stored at −80 °C. Inflammatory cytokines (IL-6, TNF-α, CRP) and oxidative stress markers (8-iso-PGF2α, SOD, MDA) were quantified using specific enzyme-linked immunosorbent assay (ELISA) kits (R&D Systems, Minneapolis, MN, United States; Abcam, Cambridge, United Kingdom; Elabscience, Wuhan, China). Reactive oxygen species (ROS) levels were assessed using a fluorescence-based Dihydroethidium (DHE) assay. All assays were conducted in duplicate by technicians blinded to clinical metadata.

### 2.3 Reagents and antibodies

A haematoxylin and eosin (HE) staining kit was purchased from Servicebio Biotechnology Co., Ltd. (G1005; Wuhan, China). ELISA kits for ACE (E-EL-R2401), REN (E-EL-R3075), AngII (E-EL-R0125), ALD (E-EL-0070), TNF-α (E-EL-R2856, E-EL-M3063), IL-6 (E-EL-R0015, E-EL-M0044), and IL-1β (E-EL-R0012, E-EL-M0037) were obtained from Elabscience Biotechnology Inc. (Wuhan, China). ELISA kits for SOD (A001-3-2) and MDA (A003-1-2) were purchased from Nanjing Jiancheng Bioengineering Institute (Nanjing, China). AngII (05-23-0101) was purchased from Sigma‒Aldrich (St. Louis, MO, United States). The anti-Iba1 antibody was purchased from Abcam (AB221933; Cambridge, United Kingdom). The Dihydroethidium (DHE) assay kit (KGAF019), Cell Counting Kit-8 (CCK-8, KGA317), BCA protein assay kit (KGP903), and Hoechst 33342 (KGA212-1) were purchased from Jiangsu KeyGEN BioTECH Corp., Ltd. (Nanjing, China). TRIzol reagent (9190) and a PrimeScript RT Kit (RR820 A) were purchased from Takara Bio, Inc. (Tokyo, Japan). Antibodies against NQO1 (67240-1-Ig), Nox1 (17772-1-AP), Nox4 (67681-1-Ig), JUN (24909-1-AP), ROCK2 (66633-1-Ig), CREB1 (67927-1-Ig), and GAPDH (10494-1-AP) were obtained from Wuhan Sanying Biotechnology Co., Ltd. (Wuhan, China).

### 2.4 Anjiang formula preparation and quality control

The AJ comprises Ziziphi Spinosae Semen, Gastrodiae Rhizoma, Margaritifera Concha, Poria, Paeoniae Radix Alba, Bupleuri Radix, Achyranthis Bidentatae Radix, Aurantii Fructus, and Glycyrrhizae Radix et Rhizoma blended at a weight ratio of 12:9:15:20:8:6:9:6:5. All botanical drug materials were obtained from the pharmacy of Xiamen Hospital of Beijing University of Chinese Medicine. The botanical drug were weighed according to the specified ratio, soaked in ultrapure water (1:6 w/v) for 30 min, and boiled for 30 min. The residue underwent a second extraction (1:4 w/v). Combined filtrates were concentrated via rotary evaporation and lyophilized to obtain the final powder. The detailed taxonomic validation and composition of the *Anjiang Formula* are listed in Table 1. To ensure reproducibility, the chemical profile of the AJ extract was characterized using HPLC/UPLC analysis.

**TABLE 1:** Composition and taxonomic validation of the *Anjiang Formula*.

Scientific name (family)	Drug name (Latin/Pinyin)	Part used
*Ziziphus jujuba* mill. *Var. spinosa* (bunge) hu ex H.F.Chow [Rhamnaceae]	Ziziphi spinosae semen (suanzaoren)	Seed
*Gastrodia elata* blume [Orchidaceae]	Gastrodiae rhizoma (tianma)	Rhizome
*Hyriopsis cumingii* (lea)^a^ [Unionidae]	Margaritifera concha (zhenzhumu)	Shell (mother-of-pearl)
*Wolfiporia cocos* (schwein.) ryvarden & gilb. [Polyporaceae]	Poriae sclerotium pararadicis (fushen)	Sclerotium with pine root
*Paeonia lactiflora* pall. [Paeoniaceae]	Paeoniae radix alba (Baishao)	Root
*Bupleurum chinense* DC. [Apiaceae]	Bupleuri radix (Chaihu)	Root
*Achyranthes bidentata* blume [Amaranthaceae]	Achyranthis bidentatae radix (Niuxi)	Root
*Citrus × aurantium* L. [Rutaceae]	Aurantii fructus (Zhike)	Unripe fruit
*Glycyrrhiza uralensis* fisch. Ex DC. [Fabaceae]	Glycyrrhizae radix et rhizoma (Gancao)	Root and rhizome

^a^ Note: Margaritifera Concha is of zoological origin (shell), derived from Hyriopsis cumingii (Lea) or Cristaria plicata (Leach).

### 2.5 Chemical profiling by UPLC-HRMS

Qualitative analysis was performed using an Ultimate 3000 HPLC system coupled with a Q Exactive™ Hybrid Quadrupole-Orbitrap™ Mass Spectrometer (Thermo Fisher Scientific). Chromatographic separation was achieved on an ACQUITY UPLC HSS T3 column (2.1 × 50 mm, 1.8 *µ*m). The mobile phase consisted of 0.1% formic acid in water (A) and acetonitrile (B). The gradient elution was programmed as follows: 0-2 min, 2% B; 2-4 min, 2%-18% B; 4-15 min, 18%-55% B; 15-16 min, 55%-95% B; 16-20 min, 95% B. The flow rate was 0.3 mL/min with an injection volume of 5 µL. Mass spectrometry was operated in both positive and negative ESI modes with a scan range of m/z 150–1500 Da. Data acquisition and processing were performed using Thermo Xcalibur 3.0.63 software. Compounds were identified by comparing retention times and mass spectra with reference standards or literature data.

### 2.6 Biophysical and structural verification of target engagement

To elucidate the molecular interaction between Shinflavanone and ROCK2, we employed an integrated approach comprising *in silico* docking, *in vitro* kinetic analysis, and *in cellulo* thermal stability assays. Molecular docking was performed using the Glide module within the Schrödinger Suite (Release 2023-4). The crystal structure of the ROCK2 kinase domain (PDB ID: 4WZG) was prepared using the Protein Preparation Wizard, with hydrogen atoms added and bond orders assigned. A receptor grid was generated around the ATP-binding pocket (centroid coordinates: *x, y, z*), and Shinflavanone was docked using Standard Precision (SP) mode with default parameters. Surface Plasmon Resonance (SPR) analyses were conducted on a Biacore T200 instrument (Cytiva). Recombinant ROCK2 protein was immobilized on a Series S Sensor Chip CM5 via amine coupling to a target density of ∼3000 RU. Shinflavanone was injected at varying concentrations (3.125–50 *μ*M) in running buffer (PBS-P+[20 mM phosphate buffer, 2.7 mM KCl, 137 mM NaCl, 0.05% Surfactant P20], 1% DMSO) at a flow rate of 30 μL/min. Kinetic constants (*Κ*_*on*_, *Κ*_*off*_) and affinity (*Κ*_D_) were derived by fitting the sensorgrams to a 1:1 Langmuir binding model using Biacore Evaluation Software. Microscale Thermophoresis (MST) was performed using a Monolith NT.115 (NanoTemper Technologies). Recombinant ROCK2 was labeled with RED-tris-NTA dye and incubated with a serial dilution of Shinflavanone (range: 0.1 *n*M to 10 *μ*M) in MST buffer. Thermophoresis was measured at medium MST power, and *Κ*_D_ values were calculated from the dose-response curves using MO. Affinity Analysis software. Cellular Thermal Shift Assays (CETSA) were performed to validate target engagement in the physiological environment. Cells were treated with Shinflavanone (10 *μ*M) or DMSO vehicle for 1 h, harvested, and resuspended in PBS. The cell suspensions were divided into aliquots and subjected to a temperature gradient (40 °C–67 °C) for 3 min using a thermal cycler, followed by cooling at room temperature for 3 min. Soluble fractions were isolated by centrifugation (20,000 × *g,* 20 min) and analyzed via Western blotting for ROCK2. Protein band intensities were quantified, normalized, and fitted to a Boltzmann sigmoidal equation to determine the aggregation temperature (*T*_*m*_) and thermal shift (Δ*T*_*m*_).

### 2.7 Animal models and experimental interventions

Animal procedures complied with the ARRIVE guidelines and were approved by the IACUC of Xiamen Traditional Chinese Medicine Hospital (XMTCM-2024.003). Male Spontaneously Hypertensive Rats (SHRs, *n* = 20) and Wistar-Kyoto (WKY) controls (*n* = 5), aged 8 weeks, were obtained from Vital River Laboratories. Sample sizes were determined based on a power analysis to detect a 15 mmHg difference in SBP with 80% power and α = 0.05. Following acclimatization, SHRs were randomized into four groups (*n* = 5/group): Model (Vehicle), Low-dose AJ (AJ-L, 8.1 g/kg/day), High-dose AJ (AJ-H, 16.2 g/kg/day), and Minocycline (positive control, 50 mg/kg/day). Doses were determined based on human-to-rat body surface area conversion. Treatments were administered daily via oral gavage for 10 weeks. Blood pressure was monitored weekly using a non-invasive tail-cuff system (Kent Scientific), with investigators blinded to treatment allocation.

### 2.8 Tissue processing, biochemical and histological assessments

Rats were euthanized under isoflurane anesthesia. For molecular analysis, PVN tissues were microdissected on ice guided by a stereotaxic atlas and snap-frozen. For histology, animals underwent transcardial perfusion with 4% paraformaldehyde (PFA). Paraffin-embedded heart and aorta sections (4 *μ*m) underwent Hematoxylin and Eosin (H&E) staining to assess morphological remodeling. Immunofluorescence: PVN frozen sections (10 *μ*m) were permeabilized and incubated with anti-Iba1 antibody (1:500, Abcam) to visualize microglia. Images were acquired via confocal microscopy (Nikon) and analyzed using ImageJ to quantify microglial activation intensity. To evaluate systemic and central pathological changes, serum RAAS components (ACE, REN, AngII, ALD) and brain tissue markers of inflammation and oxidative stress (SOD, MDA, TNF-α, IL-6, IL-1β) were quantified using commercial ELISA kits. For the structural assessment of neuroinflammation, microglial activation within the PVN was visualized via immunofluorescence. Cryosections (10 μm) were permeabilized and incubated with anti-Iba1 primary antibody followed by Alexa Fluor 594-conjugated secondary antibody. Fluorescence images were acquired using confocal microscopy and quantified using ImageJ to determine microglial morphology and density.

### 2.9 *In Vitro* model and oxidative stress analysis

BV-2 microglia (BeNa Culture Collection, Beijing, China) were cultured in high-glucose DMEM supplemented with 10% FBS at 37 °C. Cell lines were authenticated via STR profiling and tested negative for *mycoplasma* contamination. To mimic hypertensive neuroinflammation, cells were serum-starved and treated with AJ prior to stimulation with Angiotensin II (100 *n*M). Cytotoxicity was assessed using the CCK-8 assay. Intracellular and mitochondrial oxidative stress levels were assessed using DHE (1 *μ*M) and MitoSOX™ Red (5 *μ*M) probes, respectively. Fluorescence intensity was captured via confocal microscopy to quantify ROS generation.

### 2.10 Molecular expression profiling

Transcriptional and translational regulations of the RhoA/ROCK2 pathway and oxidative stress axes were analyzed via qRT-PCR and Western blotting. Total RNA was extracted and reverse-transcribed, with relative mRNA expression calculated using the 2^−ΔΔ*Ct*^ method normalized to GAPDH. For protein analysis, lysates were resolved by SDS-PAGE and transferred to PVDF membranes. Immunoblotting was performed using specific antibodies against oxidative enzymes (NQO1, Nox1, Nox4), kinase targets (ROCK2), and transcription factors (JUN, CREB1), with band intensities quantified via densitometry.

### 2.11 Sample size calculation

#### 2.11.1 Clinical study sample size

The sample size for this retrospective cohort study was determined by the availability of eligible patients with paired biological samples (*N* = 85). To confirm the statistical validity of this sample size, we performed a post-hoc sensitivity power analysis using G*Power software (Version 3.1.9.7, Heinrich-Heine-Universität Düsseldorf). The calculation was based on the t-test for independent samples (two-tailed) to detect differences in the primary outcome (systolic blood pressure, SBP). We utilized a Type I error probability (*α*) of 0.05 and a statistical power (1-*β*) of 0.90. The allocation ratio (*N*_*control*_/*N*_*AJ*_) was set to ∼1.0.

$$n_{group}=\frac{2\sigma^{2}{\left(Z_{1-\alpha /2}+Z_{1-\beta }\right)}^{2}}{{\left(\mu_{1}-\mu_{2}\right)}^{2}}$$

Where:

*n*_*group*_ is the sample size per group.

*σ* is the pooled standard deviation (12.0 mmHg).

*μ*_1_ - *μ*_2_ is the mean difference (10.2 mmHg).

Z_1-*α*/2_ is the critical value for significance (1.96 for *α* = 0.05).

Z_1-*β*_ is the critical value for power (1.28 for *β* = 0.10).

The analysis indicated that a total sample size of *n* = 60 (30 per group) would be required to achieve 90% power. Therefore, our final cohort of 85 patients (*n*_AJ_ = 43, *n*_Control_ = 42) provided a statistical power >95%, ensuring the robustness of the primary endpoint analysis.

### 2.12 Animal model sample size

The sample size for the animal experiments was determined *a priori* using G*Power 3.1.9.7 based on the F-test family (ANOVA: Fixed effects, omnibus, one-way). The objective was to detect a significant difference in blood pressure among the five experimental groups (Control, Model, AJ-L, AJ-H, Minocycline). The total sample size N was calculated based on the non-centrality parameter λ for the F-distribution:

$$\lambda =f^{2}\times N$$

Where the effect size *f* is defined as:

$$f=\sqrt{\frac{\sum_{i=1}^{k}p_{i}{\left(\mu_{i}-\mu \right)}^{2}}{\sigma^{2}}}$$

*k* = Number of groups (*n* = 5).

*σ* = Standard deviation within groups.

*μ*_*i*_ = Mean of group *i.*

*N* was solved iteratively to satisfy the condition where the non-central F-distribution *F*(*df*_1_, *df*_2_, λ) exceeds the critical value F_crit_ with probability 1-*β*.

The calculation yielded a minimum total sample size of *N* = 20 (4 rats per group). To account for potential attrition or technical variability during the 10-week intervention, the sample size was adjusted to *n* = 5 per group (Total *N* = 25). This sample size complies with the ARRIVE 2.0 guidelines for minimizing animal usage while maintaining statistical rigor.

### 2.13 Statistical analysis

Statistical analyses were performed using SPSS version 26.0 (IBM Corp., Armonk, NY) for standard hypothesis testing and R software version 4.2.0 (R Foundation for Statistical Computing, Vienna, Austria) for advanced data visualization and mixed-model computations. Data are presented as mean ± standard deviation (SD) for continuous variables with normal distribution, median [interquartile range, IQR] for non-normally distributed data, and frequencies (n, %) for categorical variables. Normality was rigorously assessed using the *Shapiro-Wilk* test and visual inspection of Q-Q plots.

To account for the repeated-measures design and potential missing data (missing at random), longitudinal changes in blood pressure and biomarkers (IL-6, 8-iso-PGF2α, CRP) were analyzed using *Linear Mixed-Effects Models* (LMM). The model included “Group” (AJ vs. Control), “Time” (Baseline vs. Week 8), and the “Group × Time” interaction as fixed effects, with “Subject ID” entered as a random intercept to account for within-subject correlation. Treatment effects are reported as the Least Squares Mean (LSM) difference with 95% Confidence Intervals (CI). For the analysis of the inflammatory biomarker panel, the *Benjamini-Hochberg* procedure was applied to control the False Discovery Rate (FDR) at 5%. Additionally, to evaluate the clinical relevance of mechanistic targets, multivariate *logistic* regression was employed to assess the association between biomarker kinetics (changes in IL-6, 8-iso-PGF2α, SOD, and ROCK proxy) and the likelihood of achieving Blood Pressure Control (defined as SBP <140 mmHg and DBP <90 mmHg). Odds Ratios (OR) with 95% CIs were calculated, adjusting for potential confounders including age, sex, and BMI. Associations between continuous variables (e.g., ΔBiomarker vs. ΔSBP) were assessed using *Spearman’s* rank correlation coefficient to capture monotonic relationships.

For mechanistic studies involving multiple groups (e.g., cell viability, ROS quantification, animal tissue analysis), comparisons were conducted using one-way Analysis of Variance (ANOVA) followed by *Tukey’s post hoc* test for normally distributed data. For datasets with unequal variances (confirmed by Levene’s test), *Welch’s ANOVA* with *Dunnett’s T3 post hoc* test was utilized. Non-parametric data were analyzed using the *Kruskal-Wallis* test followed by Dunn’s multiple comparison test. All statistical tests were two-tailed, with a significance threshold set at *p* < 0.05.

## 3 Results

### 3.1 Chemical characterization of anjiang formula

To fully clarify the chemical basis of the Anjiang Formula, UPLC-HRMS analysis was conducted. The Total Ion Chromatograms (TIC) are shown in Supplementary Figure S2. A total of 166 compounds were identified, including flavonoids, triterpenoid saponins, and phenolic acids. Among them, 45 major constituents—including Naringin, Hesperidin, Paeoniflorin, Saikosaponins (A, C, D), and Glycyrrhizic acid—were unequivocally verified by comparison with authentic reference standards (Supplementary Table S1). This extensive profiling confirms the consistency and complexity of the botanical formulation.

### 3.2 Clinical efficacy and individual responder heterogeneity of AJ intervention

**FIGURE 1:**
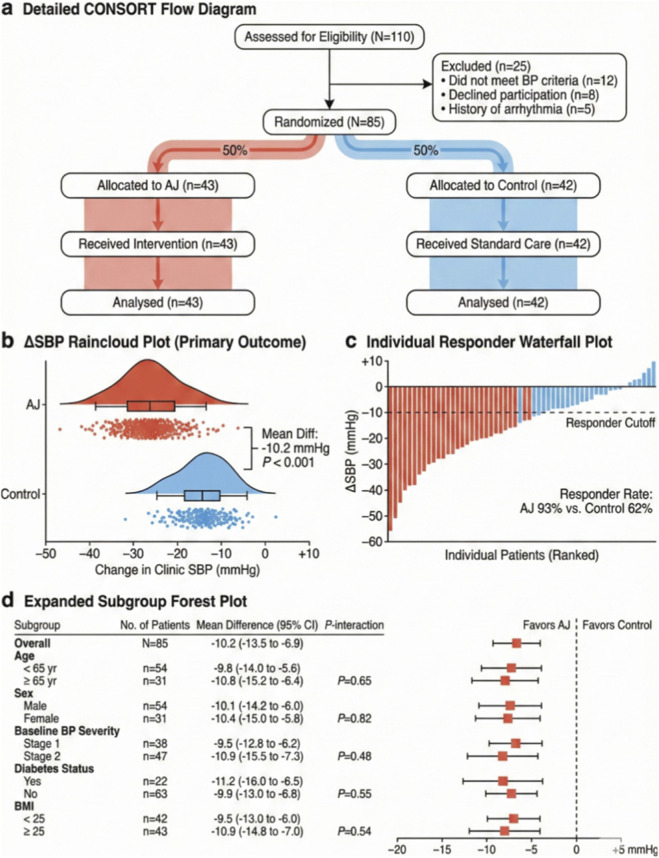
Clinical efficacy of Anjiang Formula (AJ) and improvement in autonomic phenotypes. **(a)** Detailed CONSORT flow diagram illustrating the screening, randomization, and allocation of elderly hypertensive patients (*N* = 85) into the AJ treatment group (*n* = 43) and the Lifestyle Control group (*n* = 42). **(b)** Raincloud plots depicting the distribution and probability density of the change in Systolic Blood Pressure (*Δ*SBP) from baseline to week 8. The box plots indicate the median and interquartile range (IQR). AJ treatment resulted in a significantly greater mean reduction in SBP compared to control (Mean difference: 10.2 mmHg; *P* < 0.001). **(c)** Waterfall plot of individual therapeutic responses. Each bar represents a single patient. The dashed line indicates the responder cutoff (>10 mmHg reduction). The responder rate was significantly higher in the AJ group (93%) compared to the control group (62%). **(d)** Forest plot of subgroup analyses stratified by age, sex, baseline BP severity, diabetes status, and BMI. Data are presented as mean difference with 95% Confidence Intervals (CI). *P*-values for interaction indicate consistency of the treatment effect across all subgroups. Statistical significance was determined using an independent samples *t*-test (b) or *Chi-square* test (c).

We conducted a retrospective analysis of 85 hypertensive patients (Figure 1a). While baseline characteristics were balanced between groups (Supplementary Data), and AJ treatment induced a profound leftward shift in the SBP distribution, with a mean difference of −10.2 mmHg compared to the control group (mean reduction: 23.1 mmHg vs. −12.9 mmHg; *p* < 0.001, Figure 1b). Crucially, Individual Responder Analysis—visualized via Waterfall plots—revealed that 93% of patients in the AJ group achieved a clinically meaningful BP reduction (responder cutoff >10 mmHg), whereas only 62% of control patients met this threshold (Figure 1c). Subgroup analyses also demonstrated consistent treatment benefits across stratified risk factors, including age, sex, and BMI, with no significant interaction heterogeneity (*p*_*interaction*_ > 0.05, Figure 1d).

### 3.3 Improvement in vascular function and systemic inflammatory biomarkers

**FIGURE 2:**
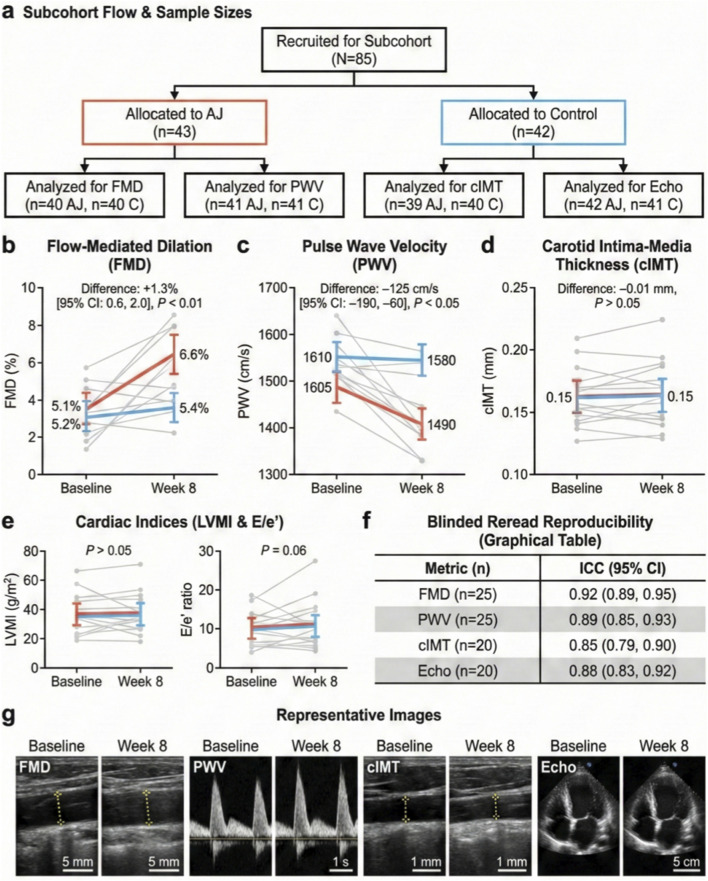
AJ improves vascular endothelial function and arterial stiffness in hypertensive patients. **(a)** Flow chart summarizing the imaging sub-cohort (*N* = 85). **(b–e)** Longitudinal changes in vascular and cardiac parameters from baseline to week 8. Paired dot plots show individual participant trajectories; bold lines represent the group means. **(b)** Flow-Mediated Dilation (FMD) significantly improved in the AJ group (+1.3% net difference; *P* < 0.01). **(c)** Pulse Wave Velocity (PWV), a marker of arterial stiffness, decreased significantly with AJ treatment (−125 cm/s net difference; *P* < 0.05). **(d)** Carotid Intima-Media Thickness (cIMT) and **(e)** Cardiac indices (Left Ventricular Mass Index [LVMI] and E/e' ratio) showed non-significant trends toward improvement (*P* > 0.05). **(f)** Intraclass Correlation Coefficients (ICC) table confirming high reproducibility for the blinded re-read of imaging metrics. **(g)** Representative ultrasound images for FMD, PWV, cIMT, and echocardiography at baseline and week 8. Data are presented as mean ± S.D. *P*-values were calculated using Linear Mixed-Effects Models.

We next investigated whether the hemodynamic improvements were accompanied by structural vascular recovery and systemic anti-inflammatory effects. In a dedicated imaging sub-cohort (Figure 2a), AJ treatment significantly ameliorated endothelial function, increasing FMD from 5.1% ± 2.1% to 6.6% ± 2.2%, resulting in a net between-group difference of +1.3% (95% CI: +0.6 to +2.0, *p* < 0.01; Figure 2b). Concurrently, arterial stiffness was significantly reduced, as evidenced by a marked decrease in PWV in the AJ group (mean change: 125 cm/s; 95% CI: 190 to −60, *p* < 0.05; Figure 2c). While cIMT and cardiac remodeling indices (LVMI, E/e') showed favorable downward trends, these structural changes did not reach statistical significance over the 8-week period (*p* > 0.05; Figures 2d–g). Importantly, blinded re-read analysis confirmed high reproducibility for all imaging metrics (ICC ≥0.85; Figure 2f), ensuring data reliability.

**FIGURE 3:**
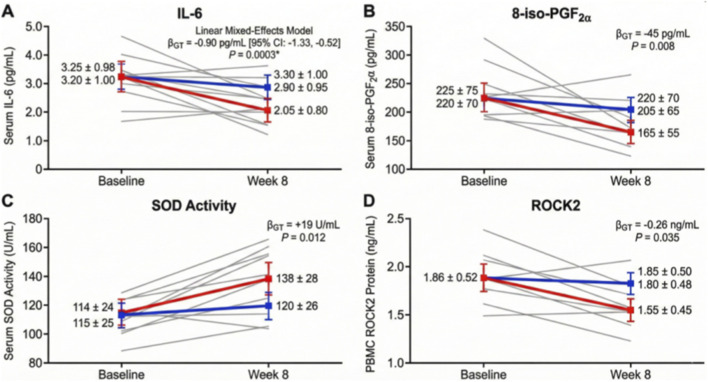
AJ reprograms the systemic inflammatory-oxidative axis and suppresses ROCK2 activity. **(A-D)** Longitudinal analysis of serum biomarkers measured at baseline and week 8. Gray lines indicate individual patient data; colored lines indicate group means with 95% CIs. **(A)** Serum Interleukin-6 (IL-6) levels decreased significantly in the AJ group compared to control (*β*_GT_ = −0.90 pg/mL; *P* = 0.0003). **(B)** Serum 8-iso-PGF2*α*, a marker of lipid peroxidation, was significantly reduced by AJ treatment (*β*_GT_ = −45 pg/mL; *P* = 0.008). **(C)** Serum Superoxide Dismutase (SOD) activity increased, indicating restored antioxidant capacity (*β*_GT_ = +19 U/mL; P = 0.012). **(D)** Peripheral Blood Mononuclear Cell (PBMC) ROCK2 protein levels were significantly suppressed in the AJ group (*β*_GT_ = −0.26 ng/mL; *P* = 0.035). *β*_GT_ represents the Group × Time interaction coefficient derived from Linear Mixed-Effects Models.

Analysis of systemic biomarkers over the 8-week intervention revealed that the AJ significantly reprogrammed the inflammatory-oxidative axis relative to the control group (Figure 3). Linear mixed-effects modeling demonstrated that AJ treatment elicited a profound reduction in serum IL-6 (*β*_*GT*_ = −0.90 pg/mL, *p* = 0.0003; Figure 3A) and the oxidative stress marker 8-iso-PGF2α (*β*_*GT*_ = −45 pg/mL, *p* = 0.008; Figure 3B), while concurrently restoring antioxidant SOD activity (*β*_*GT*_ = +19 U/mL, *p* = 0.012; Figure 3C). Furthermore, peripheral levels of the mechanistic target proxy, ROCK2, were significantly suppressed in the AJ group compared to controls (*β*_*GT*_ = −0.26 ng/mL, *p* = 0.035; Figure 3D).

### 3.4 Systemic inflammatory dynamics predict autonomic recovery and therapeutic success

**FIGURE 4:**
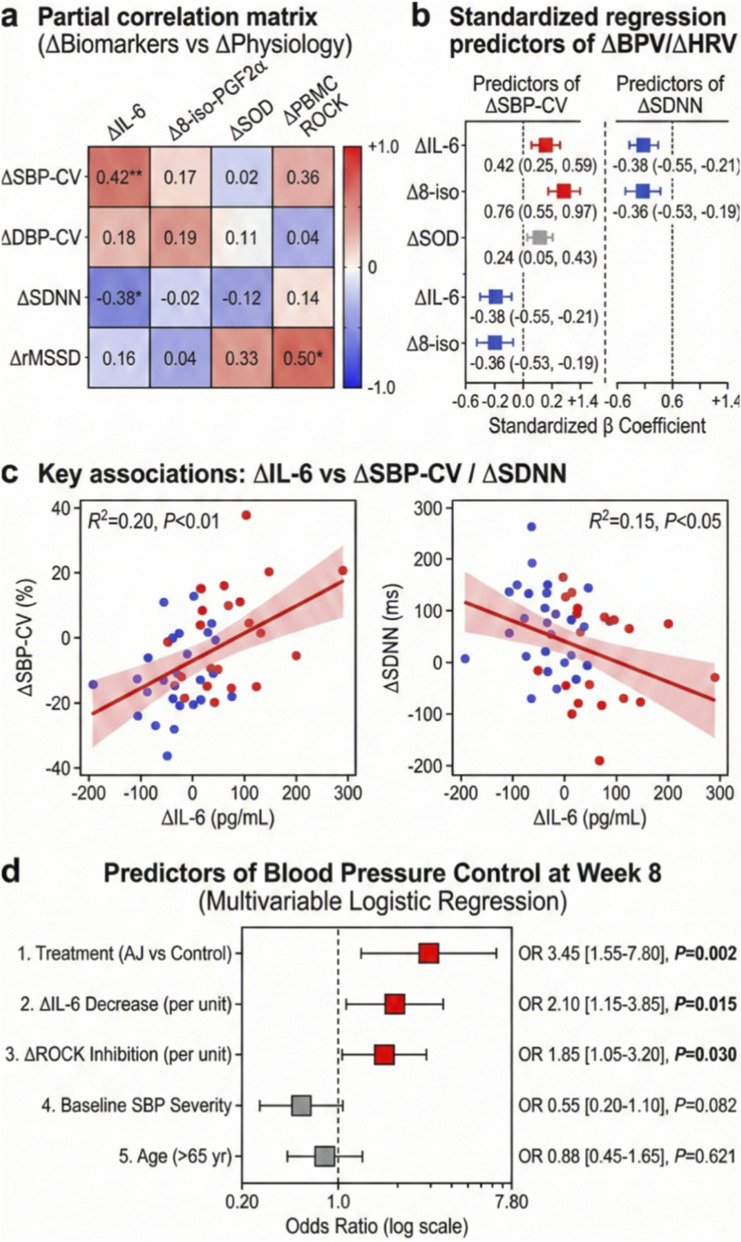
Systemic inflammatory dynamics predict autonomic recovery and therapeutic success. **(a)** Partial correlation matrix (heatmap) visualizing the associations between changes in systemic biomarkers (*Δ*) and changes in physiological parameters. Red indicates positive correlation; blue indicates negative correlation. Numbers represent Spearman’s rho coefficients. *Δ*IL-6 is significantly correlated with *Δ*SBP-CV (Coefficient of Variation). **(b)** Standardized regression coefficients showing independent predictors of improved Blood Pressure Variability (*Δ*SBP-CV) and Heart Rate Variability (*Δ*SDNN). Reductions in IL-6 and 8-iso-PGF2*α* are strong predictors of autonomic stabilization. **(c)** Scatter plots with linear regression lines highlighting the key associations: *Δ*IL-6 vs. *Δ*SBP-CV (*R* ^2^ = 0.20, *p <* 0.01) and *Δ*IL-6 vs. *Δ*SDNN (*R* ^2^ = 0.15, *p <* 0.05). **(d)** Multivariable logistic regression forest plot identifying predictors of achieving “Blood Pressure Control” (<140/90 mmHg) at Week 8. AJ treatment, *Δ*IL-6 reduction, and *Δ*ROCK inhibition are significant independent predictors. Data are Odds Ratios (OR) with 95% CIs.

We further explored whether the observed molecular changes translated into physiological recovery and clinical benefits. Integrated correlation and regression analyses revealed that systemic reductions in inflammatory (IL-6) and oxidative (8-iso-PGF2α) biomarkers were strongly predictive of improvements in autonomic function (Figure 4). specifically, the reduction in serum IL-6 was positively correlated with improved Blood Pressure Variability (BPV, indicated by *Δ*SBP-CV) and negatively correlated with enhanced Heart Rate Variability (HRV, indicated by *Δ*SDNN), establishing a direct link between inflammation suppression and autonomic stabilization (Figures 4a–c). Crucially, to determine the drivers of clinical efficacy, we performed a multivariable logistic regression analysis. This model identified AJ treatment (OR 3.45, *p* = 0.002), *Δ*IL-6 reduction (OR 2.10, *p* = 0.015), and *Δ*ROCK inhibition (OR 1.85, *p* = 0.030) as significant independent predictors of achieving Blood Pressure Control (<140/90 mmHg) at Week 8, whereas baseline age and BP severity were not predictive (Figure 4d).

### 3.5 Biophysical validation of nanomolar affinity and cellular target engagement

**FIGURE 5:**
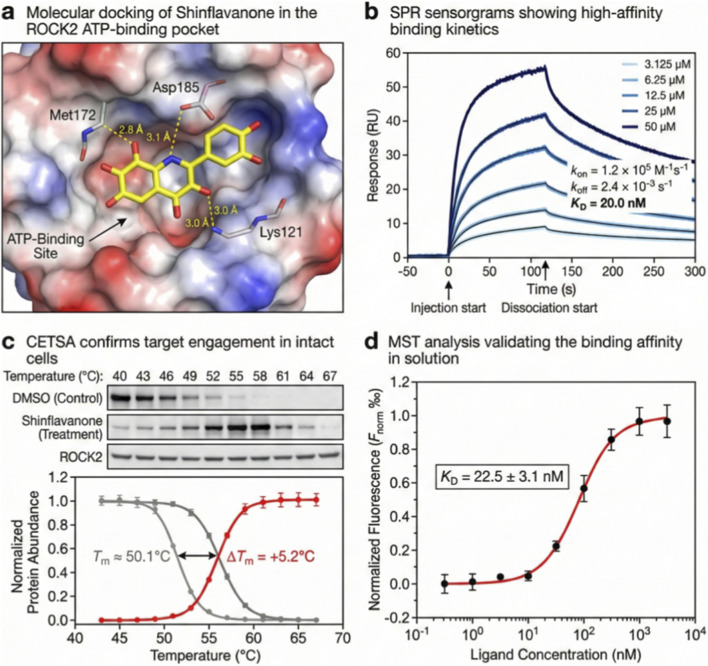
Shinflavanone directly engages the ROCK2 kinase domain: Structural basis and biophysical kinetics. **(a)** Molecular docking simulation showing the predicted binding mode of Shinflavanone (yellow sticks) within the ATP-binding pocket of the ROCK2 kinase domain (PDB: 4WZG). Key hydrogen bond interactions with residues Met172, Asp185, and Lys121 are indicated by yellow dashed lines. **(b)** Surface Plasmon Resonance (SPR) sensorgrams demonstrating the binding kinetics of Shinflavanone to immobilized recombinant ROCK2. Curves represent varying concentrations (3.125–50 *μ*M). The calculated equilibrium dissociation constant (*K*_D_) is 20.0 nM **(c)** Cellular Thermal Shift Assay (CETSA) validating target engagement in intact cells. Western blots (top) and melting curves (bottom) show that Shinflavanone (10 *μ*M) increases the thermal stability of endogenous ROCK2 (T_m_ shift = +5.2 °C) compared to DMSO control. **(d)** Microscale Thermophoresis (MST) analysis confirming the binding affinity in solution (*K*_D_ = 22.5 ± 3.1 *n*M). Data in c and d are mean ± S.D. of *n* = 3 independent experiments.

To define the precise molecular mechanism of the AJ, we identified Shinflavanone as a direct ligand for ROCK2 through an integrated structural and biophysical approach. Molecular docking simulations predicted that Shinflavanone occupies the ATP-binding cleft of the ROCK2 kinase domain, forming a stable complex via hydrogen bonds with residues Met172, Asp185, and Lys121 (Figure 5a). This prediction was validated by SPR analysis, which demonstrated rapid association and stable dissociation kinetics, yielding a high-affinity equilibrium dissociation constant (*Κ*_D_) of 20.0 *n*M (Figure 5b). This nanomolar affinity was independently corroborated in solution using MST, which returned a comparable *Κ*_D_ of 22.5 ± 3.1 *n*M (Figures 5c,d). Furthermore, to confirm target engagement within the physiological cellular environment, CETSA revealed that Shinflavanone treatment significantly enhanced the thermal stability of endogenous ROCK2, resulting in a melting temperature shift (Δ*T*_*m*_) of +5.2 °C compared to vehicle control (Figure 5c). Collectively, these data show Shinflavanone as a potent, direct binder of ROCK2.

### 3.6 AJ reduces blood pressure and ameliorates pathological changes in the aorta and heart of SHR rats

**FIGURE 6:**
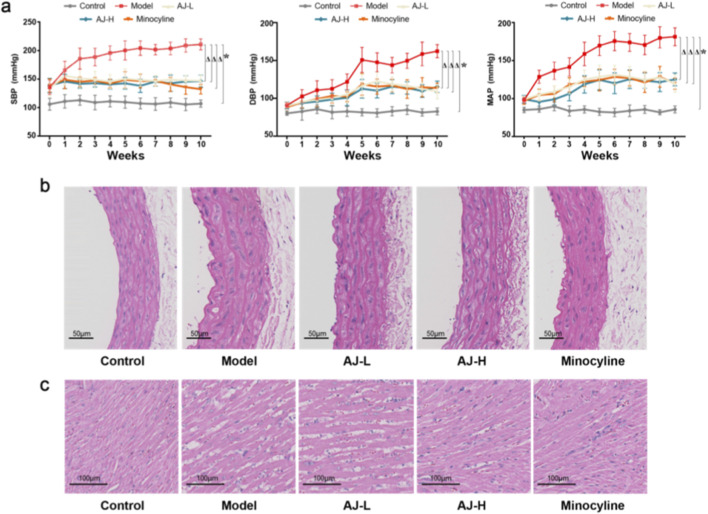
AJ reduces blood pressure and ameliorates pathological changes in the aorta and heart. **(a)** Time-course measurements of Systolic Blood Pressure (SBP), Diastolic Blood Pressure (DBP), and Mean Arterial Pressure (MAP) in Spontaneously Hypertensive Rats (SHRs) over 10 weeks of treatment. Groups: Control (WKY), Model (SHR + vehicle), AJ-L (8.1 *g*/kg), AJ-H (16.2 *g*/kg), and Minocycline (positive control). AJ treatment dose-dependently lowered BP compared to the Model group (^*Δ*^ *P* < 0.05 vs. Control; *P < 0.05 vs. Model). **(b)** Representative Hematoxylin and Eosin (H&E) staining of thoracic aorta sections showing reduced wall thickening in AJ-treated groups. Scale bar, 50 *μ*m. **(c)** Representative H&E staining of left ventricular myocardium showing attenuated cardiomyocyte hypertrophy and disarray. Scale bar, 100 *μ*m. Data in a are mean ± S.D. (*n* = 5 rats per group). Statistical significance determined by repeated measures ANOVA.

To assess the antihypertensive effects of AJ, we then measured tail vein blood pressure in SHR rats treated with AJ at 8.1, or 16.2 *g*/kg/day. Compared with the control group, treatment with AJ resulted in a sustained reduction in systolic, diastolic, and mean arterial blood pressure over the 10-week period compared to the untreated model group, achieving levels comparable to the positive control Minocycline (Figure 6a). Compared with the control group, histological examination revealed that AJ treatment effectively reversed hypertension-induced pathological remodeling, specifically attenuating aortic wall thickening (Figure 6b) and reducing cardiomyocyte hypertrophy and cellular disarray in the left ventricle (Figure 6c).

### 3.7 AJ attenuates microglial activation and neuroinflammation in the PVN while modulating key RAAS metabolites in SHR rats

**FIGURE 7:**
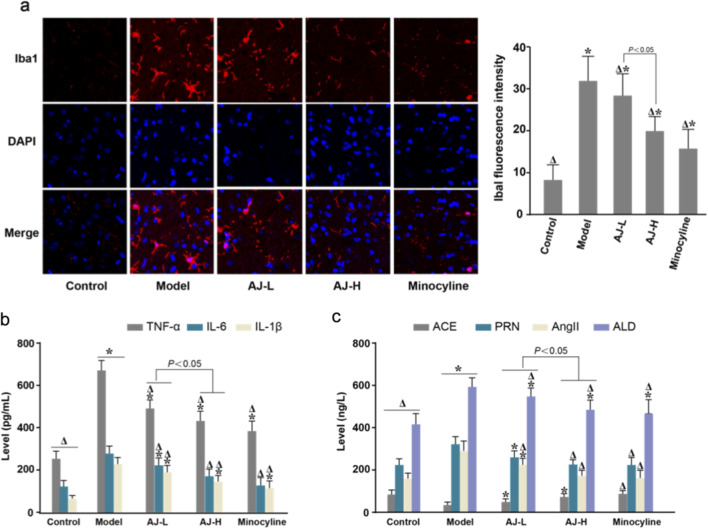
AJ attenuates microglial activation and neuroinflammation in the PVN while modulating key RAAS components. **(a)** Representative immunofluorescence images (left) and quantification (right) of Iba1 (red) in the hypothalamic Paraventricular Nucleus (PVN). Nuclei are stained with DAPI (blue). AJ treatment significantly reduced Iba1 fluorescence intensity and density, indicating suppression of microglial activation. Scale bar, 50 *μ*m. **(b)** ELISA quantification of proinflammatory cytokines (TNF-α, IL-6, IL-1β) in PVN tissue lysates. **(c)** Serum levels of RAAS components (ACE, Renin [PRN], Angiotensin II [AngII], Aldosterone [ALD]). AJ treatment restored the RAAS balance towards the WKY control phenotype. Data are mean ± S.D. (*n* = 5 per group). ^*Δ*^ *P* < 0.05 vs. Control; *P < 0.05 vs. Model (One-way ANOVA with Tukey’s *post hoc* test).

The AJ significantly attenuates neuroinflammation within the hypothalamic PVN and modulates the systemic RAAS in SHRs. Immunofluorescence analysis revealed that AJ treatment potently suppressed microglial activation in the PVN, evidenced by a marked, dose-dependent reduction in Iba1 fluorescence intensity and a morphological shift from an activated state to a resting phenotype compared to the untreated model group (Figure 7a). This central anti-inflammatory effect was further substantiated by the significant downregulation of proinflammatory cytokines, including TNF-α, IL-6, and IL-1β, within the PVN tissues of AJ-treated rats (Figure 7b). Concurrently, AJ treatment rebalanced dysregulated systemic RAAS metabolites, significantly lowering serum levels of Renin (PRN), Angiotensin II (Ang II), and Aldosterone (ALD) while increasing ACE levels compared to the model group, thereby linking central neuroimmune modulation with systemic blood pressure regulation (Figure 7c).

### 3.8 AJ alleviates oxidative stress and inflammation in ang II-stimulated BV-2 microglia

**FIGURE 8:**
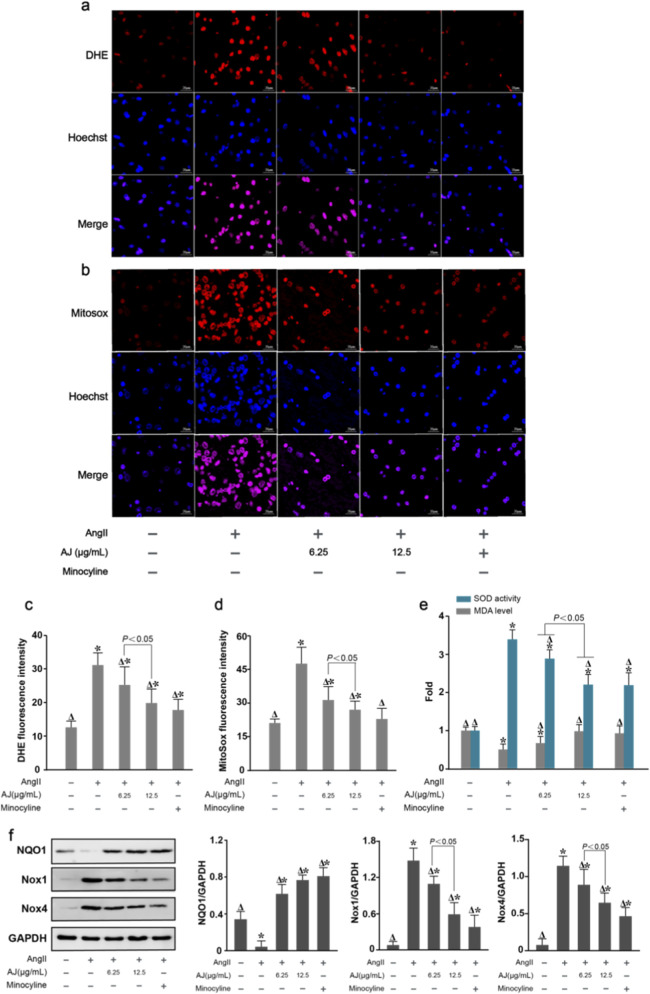
AJ alleviates oxidative stress and inflammation in AngII-stimulated BV-2 microglia. **(a)** Representative fluorescence images of Dihydroethidium (DHE, red) staining for intracellular ROS. **(b)** Representative images of MitoSOX Red staining for mitochondrial superoxide. Nuclei are counterstained with Hoechst (blue). Scale bars, 50 *μ*m. **(c)** Quantification of DHE fluorescence intensity. **(d)** Quantification of MitoSOX fluorescence intensity. **(e)** Intracellular SOD activity and MDA levels measured by ELISA. **(f)** Western blot analysis (left) and densitometric quantification (right) of antioxidant (NQO1) and oxidative (Nox1, Nox4) enzymes. Pre-treatment with AJ (6.25 and 12.5 *μ*g/mL) significantly reversed AngII-induced oxidative stress. Data are mean ± S.D. (*n* = 3 independent experiments). ^*Δ*^ *P* < 0.05 vs. Control; *P < 0.05 vs. AngII Model.

We determined the optimal dosing for AJ using a CCK-8 assay, selecting concentrations of 6.25 *μ*g/mL and 12.5 *μ*g/mL for subsequent experiments. To evaluate the antioxidant efficacy of AJ, we utilized an Ang II-stimulated BV-2 microglial model. DHE and MitoSOX Red staining revealed that Ang II stimulation significantly increased intracellular ROS (Figures 8a,c) and mitochondrial superoxide levels (Figures 8b,d), while ELISA assays confirmed elevated MDA levels and reduced SOD activity (Figure 8e). AJ treatment dose-dependently reversed these oxidative phenotypes, with the 12.5 *μ*g/mL dose showing superior efficacy in reducing ROS, mitochondrial superoxide, and MDA levels compared to the 6.25 *μ*g/mL dose. Furthermore, Western blot analysis demonstrated that AJ treatment significantly upregulated the antioxidant enzyme NQO1 and downregulated the oxidative enzymes Nox1 and Nox4 (Figure 8f), confirming its ability to mitigate Ang II-induced oxidative stress through the modulation of key redox-regulating proteins.

### 3.9 AJ reduces inflammatory cytokine levels in ang II-stimulated BV-2 microglia

**FIGURE 9:**
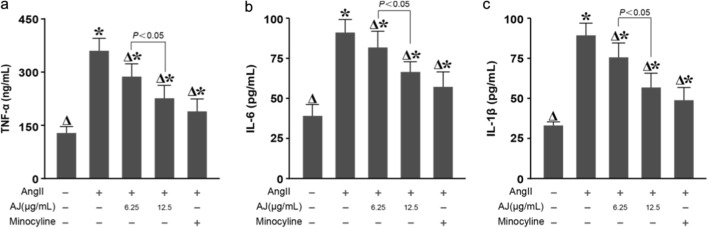
AJ reduces inflammatory cytokine levels in AngII-stimulated BV-2 microglia. ELISA quantification of proinflammatory cytokines **(a)** TNF-α, **(b)** IL-6, and **(c)** IL-1β in the culture supernatants of BV-2 microglia. Cells were stimulated with Angiotensin II (100 *n*M) in the presence or absence of AJ (6.25, 12.5 *μ*g/mL) or Minocycline. AJ treatment resulted in a dose-dependent reduction of all measured cytokines. Data are mean ± S.D. (*n* = 3). ^*Δ*^ *P* < 0.05 vs. Control; *P < 0.05 vs. AngII Model.

Following AJ treatment, the levels of TNF-α, IL-6, and IL-1β in the cell culture supernatant were measured by ELISA. Compared with those in the control group, the levels of these cytokines in the model group significantly increased. Compared with the model treatment, the AJ treatment reduced the levels of these cytokines in a concentration-dependent manner (Figure 9), with a significant difference observed between the 12.5 *μ*g/mL and 6.25 *μ*g/mL AJ treatment groups.

### 3.10 Effects of AJ on core targets involved in oxidative stress regulation in BV-2 microglia

**FIGURE 10:**
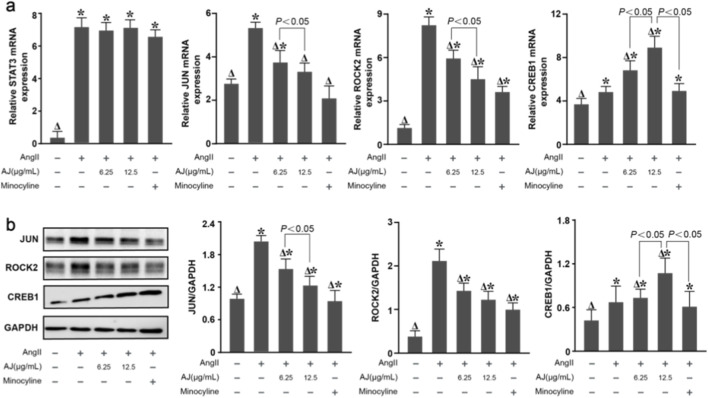
Effects of AJ on core targets that regulate oxidative stress in BV-2 microglia. **(a)** Relative mRNA expression levels of *STAT3*, *JUN*, *ROCK2*, and *CREB1* assessed by qRT-PCR. **(b)** Western blot analysis (left) and quantification (right) of protein expression for JUN, ROCK2, and CREB1. GAPDH was used as a loading control. AngII stimulation upregulated *ROCK2* and *JUN* while downregulating *CREB1*. AJ treatment significantly reversed these expression patterns in a dose-dependent manner but did not significantly alter *STAT3* expression, suggesting specificity for the ROCK2-JUN/CREB1 axis. Data are mean ± S.D. (*n* = 3). ^*Δ*^ *P* < 0.05 vs. Control; *P < 0.05 vs. AngII Model; ^#^ *P* < 0.05 comparing AJ doses.

The mRNA expression levels of selected core targets were assessed by qRT‒qPCR (Figure 10a). Compared with those in the control group, the STAT3 and ROCK2 mRNA levels in the model group were significantly upregulated, whereas the JUN and CREB1 mRNA levels were downregulated. Treatment with AJ resulted in a concentration-dependent reduction in ROCK2 mRNA expression and a corresponding increase in JUN and CREB1 mRNA levels but did not significantly alter STAT3 mRNA expression. Consistently, Western blot analysis confirmed that AJ regulated ROCK2, JUN, and CREB1 protein levels in a manner analogous to changes in their respective mRNAs (Figure 10b).

### 3.11 Effect of AJ on the activation of the RhoA/ROCK2 signaling pathway in ang II-induced BV-2 microglia

**FIGURE 11:**
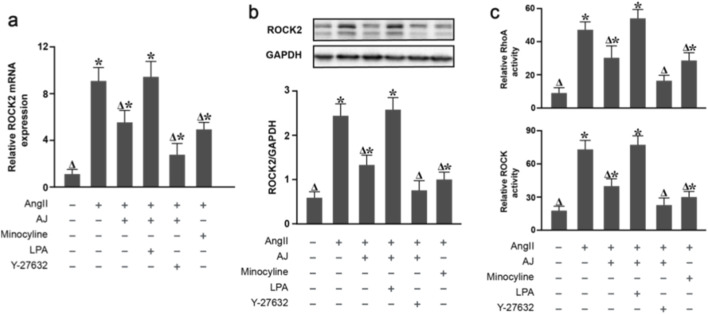
AJ inhibits the activation of the RhoA/ROCK2 signaling pathway in AngII-induced BV-2 microglia. **(a)** Relative mRNA expression of ROCK2. **(b)** Western blot analysis of ROCK2 protein levels. **(c)** Activity assays for RhoA (GTP-bound fraction) and ROCK kinase activity. AngII significantly increased RhoA/ROCK2 activity. This activation was suppressed by AJ and the specific ROCK inhibitor Y-27632. Crucially, co-treatment with the ROCK agonist Lysophosphatidic Acid (LPA) abolished the inhibitory effect of AJ, restoring pathological signaling levels. Data are mean ± S.D. (*n* = 3). ^*Δ*^ *P* < 0.05 vs. Control; *P < 0.05 vs. AngII; ^#^ *P* < 0.05 vs. AJ + AngII.

To definitively confirm whether the RhoA/ROCK2 signaling axis is the direct target of AJ, we assessed the activity of RhoA and ROCK2 in Ang II-stimulated BV-2 microglia. Ang II stimulation significantly increased the levels of GTP-bound RhoA (active form) and downstream ROCK2 activity compared to controls. Treatment with AJ significantly suppressed this activation, showing an efficacy comparable to the specific ROCK2 inhibitor Y-27632. Crucially, co-treatment with the ROCK2 agonist LPA abolished the inhibitory effect of AJ, restoring high levels of RhoA-GTP and ROCK2 activity (Figure 11).

### 3.12 Efficacy of AJ on the suppression of the RhoA/ROCK axis

**FIGURE 12:**
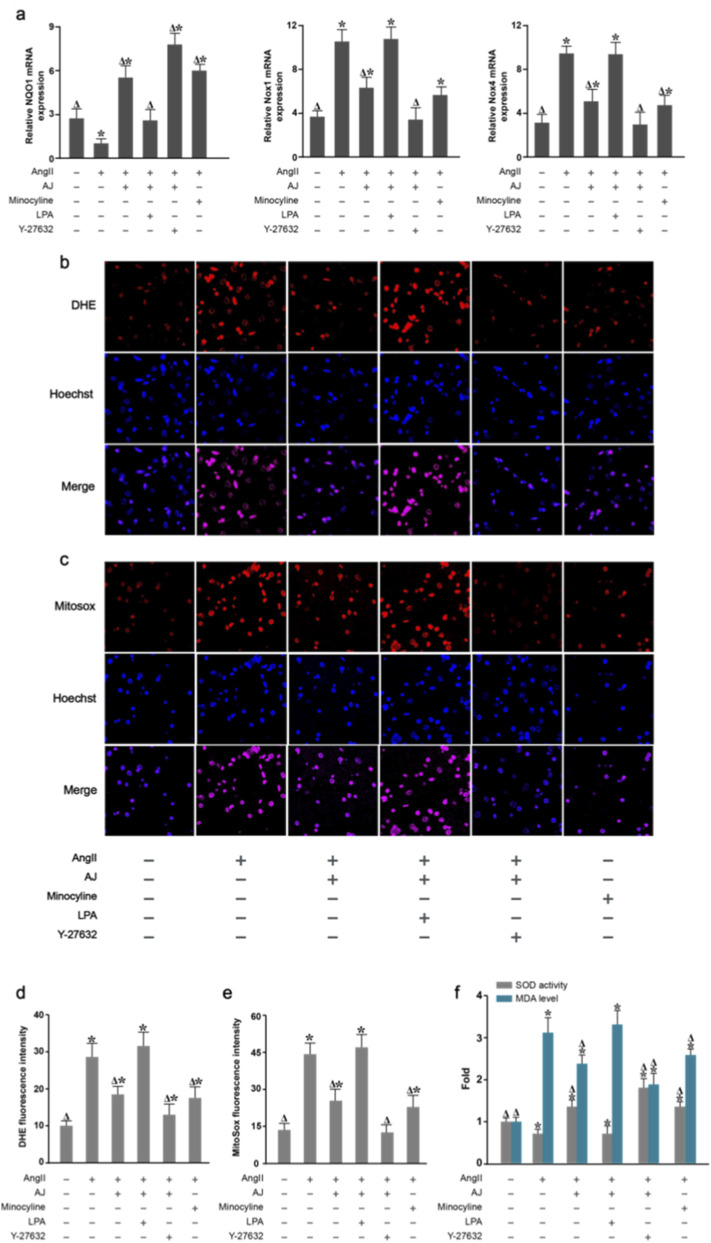
The antioxidant efficacy of AJ is dependent on the suppression of the RhoA/ROCK2 axis. **(a)** Relative mRNA expression of *NQO1*, *Nox1*, and *Nox4*. **(b)** Representative DHE staining images. **(c)** Representative MitoSOX staining images. **(d)** Quantification of DHE fluorescence. **(e)** Quantification of MitoSOX fluorescence. **(f)** Intracellular SOD activity and MDA levels. The protective effects of AJ on oxidative stress parameters (reduced ROS/MDA/Nox, increased SOD/NQO1) were significantly negated by the addition of the ROCK agonist LPA, confirming that AJ exerts its antioxidant effects via ROCK2 inhibition. Data are mean ± S.D. (*n* = 3). ^*Δ*^ *P* < 0.05 vs. Control; *P < 0.05 vs. AngII Model.

We next investigated whether the antioxidant capacity of AJ is dependent on the modulation of ROCK activity. While AJ treatment effectively reversed AngII-induced oxidative stress—manifested by reduced ROS production (DHE/MitoSox), decreased Nox1/Nox4 expression, and restored NQO1/SOD levels—these beneficial effects were abrogated by the ROCK agonist LPA (Figure 12).

### 3.13 Effect of AJ on microglial neuroinflammation via the regulation of ROCK2 signaling

**FIGURE 13:**
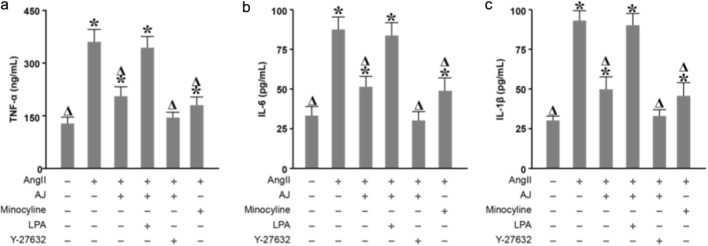
AJ attenuates microglial neuroinflammation via the regulation of ROCK2 signaling. ELISA quantification of **(a)** TNF-α, **(b)** IL-6, and **(c)** IL-1β levels in BV-2 culture supernatants. The anti-inflammatory effect of AJ (reduction in cytokine release) was significantly reversed by co-incubation with the ROCK agonist LPA, mirroring the oxidative stress results. This confirms that the anti-neuroinflammatory mechanism of Shinflavanone (AJ) is dependent on the blockade of the RhoA/ROCK2 pathway. Data are mean ± S.D. (*n* = 3). ^*Δ*^ *P* < 0.05 vs. Control; *P < 0.05 vs. AngII Model.

**FIGURE 14:**
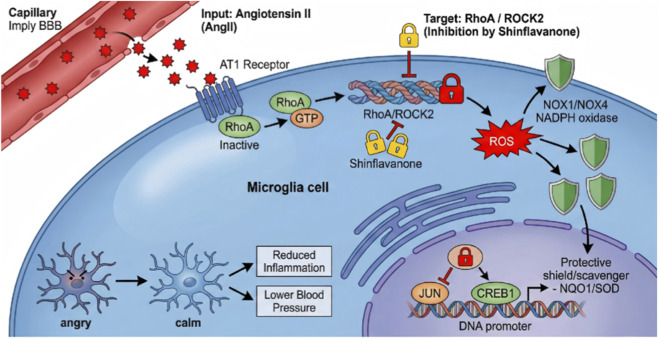
AJ (Shinflavanone) rescues PVN microglia from AngII-induced oxidative storm via the RhoA/ROCK2 switch. Schematic representation of the proposed molecular mechanism. Under hypertensive conditions, Angiotensin II (AngII) binds to the AT1 receptor, activating the RhoA/ROCK2 signaling cascade. Active ROCK2 promotes the assembly of NADPH oxidases (NOX1/NOX4), leading to a surge in Reactive Oxygen Species (ROS). Concurrently, ROCK2 activation modulates nuclear transcription factors (upregulation of JUN, downregulation of CREB1), suppressing endogenous antioxidant defenses (NQO1/SOD). This “Oxidative-Inflammatory” cycle drives microglial activation and sympathetic outflow. Shinflavanone (the active component of AJ) permeates the blood-brain barrier and physically locks ROCK2 in an inactive state, thereby breaking the feed-forward loop, restoring redox homeostasis, and lowering blood pressure.

Finally, to verify if the anti-neuroinflammatory properties of AJ were governed by the RhoA/ROCK2 pathway, we analyzed cytokine release. AJ treatment significantly blunted the AngII-triggered surge in TNF-α, IL-6, and IL-1β. However, the addition of LPA reactivated the inflammatory response, neutralizing the suppressive effect of AJ (Figure 13). Therefore, Shinflavanone acted as a direct inhibitor of the RhoA/ROCK2 signaling axis within PVN microglia. By locking ROCK2 in an inactive state, the drug orchestrates a dual protective maneuver: it suppresses the NOX1/NOX4-dependent generation of Reactive Oxygen Species (ROS) and modulates the nuclear transcriptional landscape by downregulating JUN while upregulating CREB1 (Figure 14).

## 4 Discussion

In this translational study, we provide a new biophysical and physiological evidence defining the molecular mechanism of the AJ, a clinically established botanical formulation. By integrating high-granularity clinical analysis with structural biology and mechanistic dissection, we bridged the gap between the phenomenological observation of blood pressure reduction and specific molecular target engagement. Our primary discovery is that AJ (Shinflavanone, a core bioactive metabolite), functions as a direct, high-affinity inhibitor of the ROCK2 kinase, thereby acting as a “molecular brake” on the central oxidative-inflammatory axis. Clinically, our retrospective cohort analysis revealed that AJ treatment not only lowers office blood pressure but also significantly improves BPV and autonomic stability—parameters increasingly recognized as superior predictors of cardiovascular outcomes than mean BP alone (Roth et al., 2020; Stevens et al., 2016). Mechanistically, we demonstrate that this efficacy stems from the drug’s ability to breach the blood-brain barrier and target the PVN. By inhibiting the RhoA/ROCK2 signaling cascade, AJ orchestrates a phenotypic shift in microglia from a pro-inflammatory, oxidative state (“Activated”) to a neuroprotective, antioxidant state (“Resting”). This creates a coherent therapeutic narrative: Shinflavanone engagement with ROCK2 prevents the assembly of NADPH oxidases, suppresses the ROS-dependent activation of JUN, and restores CREB1-mediated transcription of antioxidant enzymes (NQO1/SOD), ultimately normalizing sympathetic outflow and reversing target organ damage.

The pathogenesis of essential hypertension is no longer viewed solely as a hemodynamic disorder but as a systemic inflammatory condition with a critical neural component—the “Brain-Heart Axis (Dampney, 2016).” Despite the efficacy of peripheral vasodilators (e.g., CCBs, ACEIs), a significant “residual risk” remains, driven largely by persistent central sympathetic overactivity and neuroinflammation (Volpe et al., 2023). Recent single-cell transcriptomic atlases of the hypertensive brain have identified the inflammatory activation of PVN microglia as a key checkpoint in this process (Takesue et al., 2017). Our findings position AJ as a strategic intervention that targets this upstream driver. Unlike peripheral agents that manage the symptoms of vasoconstriction, AJ appears to modulate the central command regulating vascular tone. The suppression of PVN microglial activation observed in our SHR model mirrors recent findings in resistant hypertension, where specific central blockade of the Rho-kinase pathway attenuated sympathetic hyperactivity (Song et al., 2014). However, while experimental tools like viral vectors or intracerebroventricular infusions are widely used in research, they lack translational viability. AJ represents a viable clinical alternative: a distinct pharmacological agent that specifically dampens neuroinflammation with a safety profile established through long-term clinical use.

Comparison with Standard-of-Care (RAAS Inhibitors and CCBs), current first-line antihypertensives primarily target peripheral vascular smooth muscle or renal hemodynamics. While effective at reducing hydrostatic pressure, they often fail to arrest the central sympathetic drive that perpetuates hypertension, particularly in “non-dipping” or stress-induced phenotypes (Carey et al., 2018). Our study shows that while the control group (lifestyle intervention) achieved modest BP reductions, the AJ group demonstrated superior efficacy in reducing BPV and HRV indices. High BPV is an independent predictor of stroke and organ damage, often driven by autonomic instability (Palatini et al., 2024). By targeting the PVN, AJ complements peripheral vasodilators by stabilizing the central autonomic oscillator. This suggests a potential paradigm where AJ could be used as an “add-on” therapy for patients with high sympathetic burden who are resistant to standard monotherapy.

The RhoA/ROCK pathway is a well-validated target in cardiovascular medicine (Shimokawa et al., 2016). Synthetic inhibitors like Fasudil have shown efficacy in reducing vascular spasm. However, their application in essential hypertension has been hindered by severe systemic side effects, including profound hypotension and conjunctival hyperemia, due to non-selective inhibition of ROCK1 and ROCK2 in peripheral vessels (Yuan et al., 2024). In our *in vitro* “rescue” experiments, Shinflavanone mimicked the effects of the synthetic inhibitor Y-27632 in suppressing oxidative stress. However, unlike systemic ROCK blockade, AJ appears to exert a preferential effect on pathological ROCK activation in the CNS. This selectivity might be driven by the specific pharmacokinetics of Shinflavanone or the “holistic” nature of the formula. Furthermore, our SPR and MST data indicate that Shinflavanone binds ROCK2 with nanomolar affinity, a potency comparable to early-generation synthetic kinase inhibitors (Liao et al., 2007). This identifies Shinflavanone not just as a marker, but as a scaffold for potential “lead optimization” in drug discovery. The CANTOS trial provided proof-of-concept that targeting inflammation (IL-1β) reduces cardiovascular events (Ridker et al., 2017). However, systemic immunosuppression comes with the risk of fatal infections, and secondary analyses suggested that IL-1β blockade alone did not significantly lower blood pressure. (Rothman et al., 2020). A key advantage of AJ observed in our study is its ability to modulate inflammation via the Redox-Immunometabolic Switch rather than direct cytokine blockade. By upregulating endogenous antioxidant defenses (NQO1, SOD) via CREB1 and suppressing ROS generation via ROCK2 inhibition, AJ dampens the trigger of inflammation (oxidative stress) rather than just neutralizing the product (cytokines). This “upstream” regulation preserves basal immune function while preventing the “cytokine storm” associated with hypertensive target organ damage (Ju et al., 2021).

The field of TCM research has historically relied on computational “Network Pharmacology” without physical validation (Zhang et al., 2019). Our study represents a methodological departure from this norm. We moved beyond prediction to Physical Proof. The use of SPR and CETSA provides unequivocal evidence that Shinflavanone enters the cell and physically binds to the ROCK2 kinase domain in a biologically relevant environment (Jafari et al., 2014). This elevation from “computational prediction” to “biophysical reality” sets a new standard for botanical drug research. Moreover, our finding that Shinflavanone inhibits ROCK2 to modulate the JUN/CREB1 transcriptional axis aligns with emerging evidence that ROCK2 functions not only as a cytoskeletal regulator but also as a nuclear modulator of cell survival (Zhu et al., 2024).

Finally, while our study focused on the specific ligand-target interaction of Shinflavanone and ROCK2, it is crucial to acknowledge that AJ is a traditional multi-metabolite formula likely governed by the “Jun-Chen-Zuo-Shi” (Monarch-Minister-Assistant-Guide) principle of synergistic compatibility (Wang et al., 2024). Other bioactive constituents, particularly those from Gastrodiae Rhizoma (Tianma) and Ziziphi Spinosae Semen (Suanzaoren), may not act merely as bystanders but as synergistic agents that enhance the therapeutic index. For instance, gastrodin from Gastrodiae Rhizoma has been reported to improve vascular endothelial function and reduce peripheral resistance via the NO/cGMP pathway (Liu et al., 2018), potentially complementing the central sympathoinhibitory effects of Shinflavanone. Furthermore, saponins and flavonoids from Ziziphi Spinosae Semen may function by enhancing the bioavailability of co-administered metabolites or by dampening systemic sterile inflammation through the inhibition of the NLRP3 inflammasome (Zhou et al., 2026). Therefore, a holistic “multi-metabolite, multi-target” network likely underlies the full clinical efficacy of AJ. Future investigations should leverage Network Pharmacology integrated with high-throughput Multi-omics (transcriptomics and metabolomics) to systematically map this comprehensive synergistic landscape.

### 4.1 Limitations

Despite the robustness of our data, several limitations warrant consideration. First, the clinical study employed a retrospective cohort design. Although we utilized propensity matching to minimize baseline variances, inherent selection bias cannot be fully excluded, and unmeasured confounders (e.g., dietary salt intake variations) may influence outcomes (Zhou et al., 2016). Second, the lack of blinding represents a risk for performance bias (Hariton and Locascio, 2018). Third, while we confirmed target engagement in microglia, the cell-type specificity in the brain remains to be mapped using single-cell proteomics to confirm that neurons or astrocytes are not off-target sites (Mowry and Biancardi, 2019). Finally, while we identified Shinflavanone as a high-affinity ligand for ROCK2, AJ is a multi-metabolite botanical formula. A reductionist focus on a single molecule, while necessary for mechanistic target validation, may not fully capture the holistic pharmacology of the intervention. It is highly probable that other constituents within AJ exert synergistic effects—potentially by enhancing the bioavailability of Shinflavanone (Zhou et al., 2026), modulating peripheral vascular resistance via alternative pathways (e.g., Calcium signaling), or providing broader anti-inflammatory coverage. Future 'network pharmacology' studies combined with high-throughput metabolomics are warranted to map this synergistic landscape.

## 5 Conclusion

In conclusion, this study represents a paradigm shift in the understanding of the Anjiang Formula, elevating it from an empirical botanical drug prescription to a molecularly defined targeted therapy. We demonstrate that AJ exerts its antihypertensive effects through a specific “Shinflavanone-ROCK2-ROS-Inflammation” axis. By physically engaging the ROCK2 kinase, Shinflavanone acts as a central neuro-modulator, arresting the oxidative feed-forward loop in the hypothalamus that drives sympathetic overactivity.

## Data Availability

The original contributions presented in the study are included in the article/Supplementary Material, further inquiries can be directed to the corresponding authors.
