# Supplementary material for: Anjiang formula inhibits PVN microglial activation and lowers blood pressure by targeting RhoA/ROCK2 pathway: a retrospective clinical and experimental study
Source: Front Pharmacol. 2026 Feb 19;17:1795297. doi: 10.3389/fphar.2026.1795297 (PMC12960113; doi:10.3389/fphar.2026.1795297)
Supplement: Supplementary file 1 [file Supplementaryfile1.pdf]

Supplementary Table 1 Active components of AJ and corresponding targets

Pharmaceutical name	Chinese name	Latin botanical name	TSCMP		HERB		Literature	
			Compounds	Targets	Compounds	Targets	Compounds	Targets
Semen Ziziphi Spinosae	酸枣仁（*Suanzaoren*）	*Ziziphi Spinosae Semen*	9	149			1	3
Tall Gastrodiae	天麻（*TianMa*）	*Gastrodiae rhizoma*			22	435		
Common Achyranthes	牛膝（*NiuXi*）	*Achyranthis bidentatae radix*	20	417			2	88
Indian Buead Tuckahoe	茯神（*FuShen*）	*Poria*			9	205		
White Peony Root	白芍（*Baishao*）	*Paeoniae radix alba*	13	254			1	23
Chinese Thorawax Root	柴胡（*Chaihu*）	*Bupleuri radix*	17	376			4	15
Submature Bitter Orange	枳壳（*Zhike*）	*Aurantii Fructus*	5	218				
Licorice Root	甘草（*Gancao*）	*Glycyrrhizae radix et rhizoma*	92	786				

Supplementary Table 2 Primer sequences

Gene name	Forward primer	Reverse primer
**STAT3**	TTAACATTCTGGGCACGAACA	TGACAATCAAGGAGGCATCAC
**ROCK2**	GGTCAATCAGCTCCAGAAAC	GGTCAATCAGCTCCAGAAAC
**JUN**	ACTCGGACCTTCTCACGTC	GGTCGGTGTAGTSGTGATGT
**CREB1**	TGTACCACCGGTATCCATGC	TGGATAACTGATGGCTGGGC
**GAPDH**	GCAGGAGTACGATGAGTCCG	ACGCAGCTCAGTAACAGTCC

Supplementary Table 3 Core targets of AJ in the treatment of hypertension

Name	Degree	Betweenness	Closeness
AKT1	18	0.055635354	0.503623188
HRAS	17	0.057383823	0.503623188
PIK3R1	17	0.050420957	0.492907801
RAF1	16	0.04638694	0.486013986
EGFR	15	0.032371817	0.460264901
NFKB1	12	0.029156486	0.451298701
BRAF	11	0.020661756	0.423780488
MTOR	10	0.012926575	0.411242604
PDGFRA	10	0.012382485	0.413690476
PRKACA	10	0.024877779	0.437106918
SRC	10	0.010653939	0.401734104
FGF2	9	0.013192358	0.418674699
FGFR1	9	0.012050147	0.413690476
IGF1R	9	0.007212976	0.390449438
JUN	9	0.017124127	0.429012346
PDGFRB	9	0.010820262	0.411242604
STAT3	9	0.012360674	0.406432749
CASP3	8	0.015250267	0.399425287
FGFR3	8	0.008798454	0.404069767
IL6	8	0.008304554	0.383977901
MET	8	0.008606805	0.404069767
JAK2	7	0.00960388	0.404069767
CREB1	7	0.006444456	0.379781421
F2R	6	0.007387922	0.394886364
ROCK2	6	0.007520186	0.390449438

Supplementary Table 4 Key active ingredients of AJ in the treatment of hypertension

**Active Ingredients**	**Degree Value**	**Source Herb**	**Structure**	**BBB permeament**
MOL012542<br>β-ecdysterone	35	NiuXi（*Achyranthis bidentatae radix*）	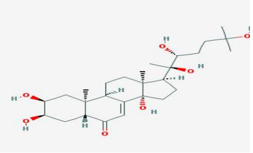	NO
MOL002714<br>baicalein	33	NiuXi（Achyranthis bidentatae radix）	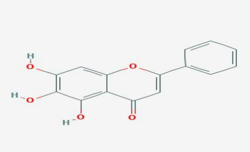	NO
MOL004598<br>3,5,6,7-tetramethoxy-2-(3,4,5-trimethoxyphenyl) chromone	31	Chaihu（*Bupleuri radix*）	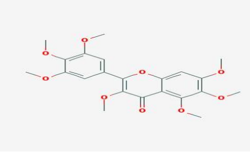	NO
MOL004824<br>(2S)-6-(2,4-dihydroxyphenyl)-2-(2-hydroxypropan-2-yl)-4-methoxy-2,3-dihydrofuro[3,2-g]chromen-7-one	31	Gancao（Glycyrrhizae radix et rhizoma）	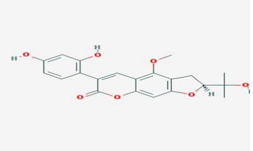	NO
MOL004961<br>Quercetin der.	31	Gancao（Glycyrrhizae radix et rhizoma）	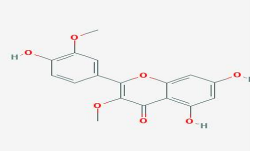	NO
MOL002897<br>epiberberine	31	NiuXi（Achyranthis bidentatae radix）	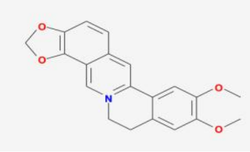	Yes
MOL004609<br>Areapillin	30	Chaihu（Bupleuri radix）	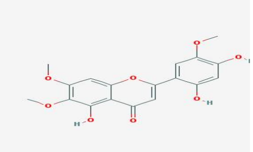	NO
MOL000239<br>Jaranol	30	Gancao（Glycyrrhizae radix et rhizoma）	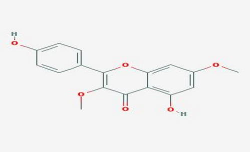	NO
MOL004805<br>(2S)-2-[4-hydroxy-3-(3-methylbut-2-enyl)phenyl]-8,8-dimethyl-2,3-dihydropyrano[2,3-f]chromen-4-one	30	Gancao（Glycyrrhizae radix et rhizoma）	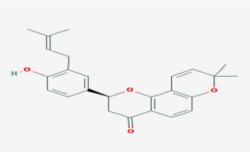	Yes

**Active Ingredients**	**Degree Value**	**Source Herb**	**Structure**	**BBB permeament**
MOL000173 wogonin	30	NiuXi（Achyranthis bidentatae radix）	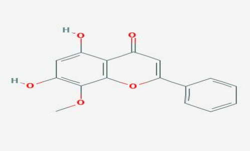	NO
HBIN010380 4-ethoxymethylphenyl-4'-hydroxybenzylether	30	TianMa（Gastrodiae rhizoma）	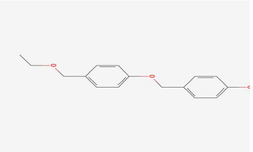	Yes
MOL000422 kaempferol	29	Baishao（Paeoniae radix alba）、Chaihu（Radix bupleuri）、Gancao（Glycyrrhizae radix et rhizoma）、NiuXi（Achyranthis bidentatae radix）	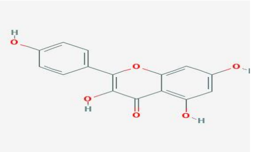	NO
MOL000354 isorhamnetin	29	Chaihu（Bupleuri radix）、Gancao（Glycyrrhizae radix et rhizoma）	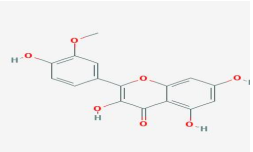	NO
MOL004949 Isolicoflavonol	29	Gancao（Glycyrrhizae radix et rhizoma）	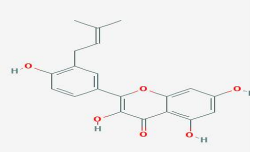	NO
MOL004941 (2R)-7-hydroxy-2-(4-hydroxyphenyl)chroman-4-one	29	Gancao（Glycyrrhizae radix et rhizoma）	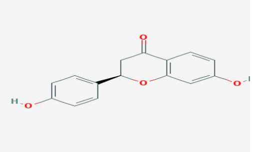	Yes
MOL000098 quercetin	28	Chaihu（Bupleuri radix）、Gancao（licorice）、NiuXi（Achyranthis bidentatae radix）	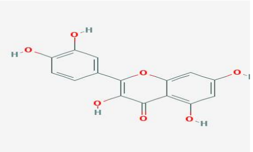	NO
MOL002565 Medicarpin	28	Gancao（Glycyrrhizae radix et rhizoma）	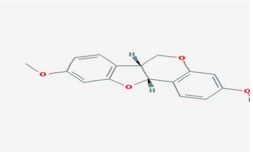	Yes
